# Stress urinary incontinence among Jordanian women living in rural areas: Prevalence, associated factors and self-management behaviours

**DOI:** 10.1080/2090598X.2021.1926751

**Published:** 2021-05-22

**Authors:** Rami AlAzab, Reema Ahmad Alomari, Yousef S. Khader, Muntaha Gharaibeh

**Affiliations:** a Division of Urology, Department of Surgery, Faculty of Medicine, Jordan University of Science and Technology, Irbid, Jordan; b Clinical Nurse Specialist in Maternal New-born Nursing, Ibn-Hayyan Pharma, Irbid, Jordan; c Department of Community Medicine, Public Health and Family Medicine, Faculty of Medicine, Jordan University of Science and Technology, Irbid, Jordan; d Secretary General of the Jordanian Nursing Council, Jordan University of Science and Technology, Irbid, Jordan

**Keywords:** Stress urinary incontinence, urge incontinence, menopause, multiparity

## Abstract

**Objectives**: To assess the prevalence of stress urinary incontinence (SUI) among Jordanian women aged 35–65 years living in in rural areas and its associated risk factors.

**Patients and methods**: A cross-sectional study utilising a convenience sample of 1000 non-pregnant women who were recruited from healthcare centres and community settings. Participants completed a structured questionnaire that included demographic and gynaecological data, and questions about SUI. Descriptive analysis and logistic regression were used to analyse the data.

**Results**: Overall, 551 women (55.1%) reported having SUI with a duration of 37.9–47.6 months. The mean age of the women was 45.38 years and 29.6% were post-menopausal. Moreover, 40.6% were overweight, 38.9% obese, and 16.9% were smokers. Married women comprised 81.8%; with 37.1% having four to six children and 92.8% had a normal delivery, whereas 28% had a history of caesarean section. Coughing was the major precipitating factor for SUI (87.7%). In addition, 64.8% of women with SUI did not avoid activities that precipitated SUI such as housekeeping and lifting, and 78.6% did not seek any medical care. The major significant correlates for SUI were: high body mass index (odds ratio [OR] 2.506, 95% confidence interval [CI] 1.744–3.600, *P* < 0.001), being aged >50 years (OR 1.716, 95% CI 1.183–2.489, *P* = 0.004), a history of gynaecological/pelvic surgery (OR 1.631, 95% CI 1.206–2.205, *P* = 0.001), and vaginal delivery (OR 1.052, 95% CI 1.004–1.101, *P* = 0.033).

**Conclusion**: SUI prevalence among Jordanian women is high with symptoms reported in more than a half of study participants. Older age, obesity, a history of gynaecological surgery, and history of vaginal delivery were the major correlates of SUI. Public awareness is needed to identify the condition for early diagnosis and treatment of SUI.

**Abbreviations:** BMI: high body mass index; OR: odds ratio; (M)(S)(U)UI: (mixed) (stress) (urge) urinary incontinence

## Introduction

Urinary incontinence (UI) is defined as involuntary leakage of urine that affects activities of daily living, associated with to loss of self-confidence and feelings of depression and helplessness [1,2]. There are three common subtypes of UI in adult women; stress UI (SUI), urge UI (UUI) and mixed UI (MUI), which is a combination of UUI and SUI. The ICS clinically defines SUI as the involuntary leakage of urine during increased abdominal pressure, in the absence of detrusor contraction.

There are variations in the prevalence of SUI among major studies, e.g. in the Kingdom of Saudi Arabia (KSA), the prevalence rate of SUI was 36.4% in one study [3] and 50% in another study [4]. Much lower prevalence was reported in Egypt [5]. Age, obesity, vaginal birth and diabetes were reported as common risk factors of SUI in these studies [4–6].

In the present study, we aimed to evaluate the prevalence of SUI and its correlates among Jordanian women. Such data might help healthcare providers assess the burden of this condition and increase knowledge and awareness of Jordanian women about this common condition.

## Patients and methods

### Study design

This cross-sectional study was conducted in 2014 to estimate the prevalence of SUI and its associated risk factors among Jordanian women aged 35–65 years. The second part of this study was concluded in 2017, directed towards reporting the long-term psychosocial effects of SUI.

The study took place at Ministry of Health peripheral medical centres and Ministry of Social Development (MOSD) in the villages surrounding the city of Irbid in North Jordan. Data collection lasted for 3 months; January–March 2014. Women who had been diagnosed with physiological problems such as bladder dysfunction, especially overactive bladder; patients with sex and/or UUI were excluded from the study.

### Instrument

Data were collected using a questionnaire completed by the participants themselves and comprised of demographic and gynaecological/obstetric data, in addition to the Norwegian screening guidelines for SUI. The sociodemographic and anthropometric part consisted of general demographic items such as age, height, weight, marital status, smoking, and educational level. The obstetric and gynaecological history included parity, method of birth, assisted birth, post-menopausal status, hormonal therapy, and any past gynaecological or urological surgical history.

The Norwegian screening guidelines for SUI were used. The guidelines were taken from the Norwegian Uro-Gynecological Group (NUGG) UI questionnaire. It contains a number of questions regarding UI in relation to coughing, sneezing, and other related activities. For the purpose of the study, another question was added related to screening for SUI during praying, as the majority of participants were Muslims. Participants who responded by ‘No’ for all screening questions were asked not to complete the rest of the questionnaire; those who had at least one ‘Yes’ response for any of the screening questions were asked to complete the questionnaire. Women were also asked to complete a set of 12 questions regarding severity, amount, avoidance of activities, reasons for seeking/not seeking healthcare.

The questions were reviewed by experts in women’s health for cultural appropriateness. Questions were originally in English and were back-translated to Arabic language (the native language of Jordanians). Questions were then back-translated by the same expert group to English. A pilot study consisting of 30 women who met the inclusion criteria was conducted to assess clarity, readability of questions, and time needed to complete all questions. The results of the pilot study confirmed that all questions were clear to all age groups and the average time needed to complete all questions was 15 min.

The study was approved by the Institutional Review Board at Jordan University of Science and Technology, the Ministry of Health and the Ministry of Social Development. Participation in the study was voluntary. Women signed a consent form prior to data collection, which included information about the purpose of the study and confidentiality of information given. Women were asked not to disclose their identity on the questionnaire to maintain anonymity.

## Results

### Participants’ characteristics

The mean age (SD) of women was 46.87 (8.7) years. Of the 551 women with SUI, 39.2% of women were overweight and 46.3% (*n* = 255) were obese. Smoking was reported by 18.7% of women. Table 1 presents the sociodemographic and obstetric characteristics of the women with SUI.

**Table 1.** Sociodemographic characteristics and obstetric and gynaecological variables of the affected women (*N* = 551)

Sociodemographic		Obstetric and gynaecological	
Variable	*N* (%)	Variable	*N* (%)
Married	454 (82.4)	Number of birth* 4–6 >6	193 (35) 151 (27.4)
High school or less	302 (54.8)	Postmenopausal	196 (35.6)
BMI Overweight Obese	216 (39.2) 255 (46.3)	Hormone replacement	19 (3.4)
Working	228 (41.4)	Urological surgery	35 (30.7)
Smoking	103 (18.7)	Gynaecological surgery (caesarean section, assisted delivery and pelvic surgery)	169 (30.7)
		Caesarean section	146 (26.4)
Educational level High school or less Diploma Bachelor degree or more	468 (46.8) 166 (16.6) 366(36.6)	Educational level High school or less Diploma Bachelor degree or more	468 (46.8) 166 (16.6) 366 (36.6)

*including live birth or abortion.

### Prevalence of SUI

Of the 1000 women screened, 55.1% (*n* = 551) reported experiencing SUI with symptoms duration of 37.9–47.6 months. In the majority of women who reported SUI and urinary leakage, cough was the commonest precipitating factor (87.7%, *n* = 483), followed by sneezing (78.5%, *n* = 432), laughing (50.5%, *n* = 278), lifting heavy objects (30.1%, *n* = 164), and 29.1% (*n* = 160) reported urinary leakage during praying.

### Severity of SUI

About 37.2% of the women with SUI reported experiencing SUI more than once daily. The extent of leakage varied from drops (54.1%, *n* = 298) to that requiring protective pads (45%, *n* = 303), while 16.9% (*n* = 93) used more than one pad a daily. Around 64.8% (*n* = 357) did not avoid activities that induced SUI like lifting, housekeeping and the remaining 35.2% (*n* = 194) occasionally avoided certain activities (11.1%, *n* = 61). The majority of the women did not seek any SUI treatment (78.6%; *n* = 433). Regarding the type of medical care the remaining 21.4% (*n* = 118) sought, 10.9% (*n* = 60) indicated consulting a gynaecologist, 6% (*n* = 33) had consulted a urologist, and 4.4% (*n* = 24) had consulted a general physician.

The reasons for not seeking medical advice were the belief that SUI was accepted as part of being a female (34.7%, *n* = 191), feeling shy about being examined (11.8%, *n* = 65) and not being bothered by the SUI symptoms (11.6%, *n* = 64), to avoid surgical procedures (3.4%, *n* = 19), the amount of leakage was manageable (7.3%, *n* = 40), and because the subject wished to avoid vaginal examination (3.8%, *n* = 21).

Affected women managed their conditions by constantly changing underwear (21.6%, *n* = 119), using protective pads (13.8%, *n* = 76), drinking herbs (11.3%, *n* = 62), avoiding soda beverages (7.3%, *n* = 40), and applying topical remedies, e.g. cream or olive oil, on the external genitalia (4.5%, *n* = 25).

### Risk factors associated with SUI

Multivariate logistic regression analysis (Table 2) showed that a high body mass index (BMI), older age, history of gynaecological surgery and history of vaginal delivery were significantly associated with SUI. Table 2 presents the multivariate logistic regression analysis of factors associated with SUI.

**Table 2.** Results of logistic regression analysis of risks factors associated with SUI

Variable	*P*	OR (95% CI)
Age, years 40–49 >50	0.003 0.002 0.004	1.662 (1.213–2.278) 1.716 (1.183–2.489)
Vaginal birth	0.033	1.052 (1.004–1.101)
Gynaecological surgery	0.001	1.631 (1.206–2.205)

OR: odds ratio. *P* value is significant at level < 0.05.

## Discussion

In the present study, we found a high prevalence of SUI among Jordanian women, with more than a half of the study participants reporting a degree of SUI. The high prevalence of SUI among Jordanian is consistent with reports from the USA, UK, Asia and Germany [7–10], and in other studies from Arab countries such as the KSA, Egypt and Jordan [3,6]. This high prevalence should be construed in light of the mean age of the study population. The mean age of our study’s participants was 46.8 years, which partially explain our findings, considering that age is one of the most important risk factors for SUI [3,7,8,10].

Age, obesity, parity, vaginal birth and gynaecological surgery were significantly associated with SUI. These factors were found to be significant risk factors in other studies in women [7–14]. Unlike other studies [3,7,8,10], assisted birth, post-menopausal status, and urological surgery were not found to be associated with SUI in our present study. This finding may be influenced by the small number of women experiencing these problems.

Our present study showed that one-third of women with SUI did not seek medical care for various reasons. First, a significant number of women in the present study perceived SUI as a normal process for being a female, highlighting the importance for raising the awareness of these symptoms as a disease that can be treated with behavioural changes, pelvic floor muscle exercises, medications, and surgery in severe cases [15]. The mistaken beliefs about SUI may be explained by the fact that the women in the present study were in their middle age and had a low level of education (educational level of 46.8% [*n* = 468] of the participants was of high school or less, and 36.6% (*n* = 366) had bachelor degree or more).

This finding is supported by previous findings on barriers to health seeking behaviour in which women considered it a normal part of ageing process [16].

Women in the present study were shy to seek medical advice, which can be explained by the percentage of women aware of the need for vaginal examination, which is considered as a very private procedure especially if done by a male physician. Other women thought that there was no need to seek help because SUI did not seem to bother them or their life that much. Women have to understand that treatment and health interventions are needed because mild symptoms might progress and adversely affect their quality of life and future treatment [17].

Regardless of the severity of symptoms, women need be informed about treatment options and the consequences of not seeking any treatment. Knowing that the majority of women in the present study were married and their marital relationship might be adversely affected by SUI, husbands could be a target for more awareness and could play a significant role in encouraging their wives to seek medical attention [18].

Some women with SUI did not perform any manoeuvres or life-style modifications to address this problem, while others reported a range of interventions, e.g. frequently changing their underwear, drinking herbal remedies, using protective pads, avoiding soda drinks, and applying topical remedies on the external genitalia. These practices reflect a lack of appropriate knowledge of SUI. These findings reciprocate those reported from Qatar, which revealed that respondents decreased fluid intake, changed underwear frequently, and used protective pads [19].

The present findings are relevant for healthcare professionals working with women. Physicians and nurses need to acknowledge the prevalence of SUI, its associated factors and practices to overcome SUI symptoms. Therefore, this topic needs to be part of nurses and physicians taught curricula to help them assess women of all ages for such problems and to fill this knowledge gap. They need to determine the modifiable risk factors and encourage women to make life-style modifications such as reducing weight, quitting smoking, and also teach women pelvic floor muscle exercises, bladder training and fluid and diet management as preventive and treatment measures. In addition, women need to understand the importance of seeking healthcare early to receive the appropriate treatment. We noticed that there was a delay in seeking medical attention in addition to consulting physicians trained to handle this condition. We acknowledge that there are some limitations of our present study. First, the cross-sectional design of this study precludes concluding a cause–effect relationship in addition to other biases introduced by this design. Second, the sampling methods of our study might not be representative of Jordanian women. Future studies with more robust methodology are encouraged to accurately identify the prevalence of SUI. Third, the diagnosis of SUI was based on patient-reported symptoms with no clinical data or diagnostics such as radiology or urodynamic findings. Moreover, other types of UI were not assessed, which might overlap or affect SUI.

## Conclusion

The prevalence of SUI among Jordanian women is high and associated with many modifiable correlates such as weight and parity. Women need to be empowered by evidenced knowledge and good practices to modify their risk factors.
